# Supplementary material for: Associations with HIV preexposure prophylaxis use by cisgender female sex workers in two Ugandan cities
Source: PLoS One. 2025 Mar 20;20(3):e0320065. doi: 10.1371/journal.pone.0320065 (PMC11925456; doi:10.1371/journal.pone.0320065)
Supplement: Attachment — Submitted filename: Response to Reviewers.doc [file pone.0320065.s003.doc]

**Makerere	University**

P.O. Box 7072 Kampala, Uganda	**Tel:** 256 414 530404

**E-mail**: [nursing@chs.mak.ac.ug](mailto:nursing@chs.mak.ac.ug)

**SCHOOL OF HEALTH SCIENCES**

**Department of Nursing**

Dr. Hamufare D Mugauri

Academic Editor, Plos One

December 16^th^, 2024

Dear Dr. Hamufare,

Regarding PONE-D-24-24744

We are very grateful for the reviewer comments provided by the reviewers of our manuscript: “*Associations with HIV Preexposure Prophylaxis Use by Cisgender Female Sex Workers in Two Ugandan Cities ".* Reviewer comments are encouraging, and the reviewers appear to share our view that study findings are of public health importance. The suggestions offered by the reviewers have been greatly helpful. Please find below our point-by-point responses in italics, with new text in bold font:

**Reviewer Comments**

**Reviewer 1**

1. Within the first paragraph of the introduction, please be more specific with terms such as "successful uptake" and "highly effective solution." Please provide greater background on PrEP, its efficacy, and context within Uganda.

*Response: We thank the reviewers for this observation. We apologize for the choice of words like successful, and highly effective solution that portrayed PrEP to be 100% effective or a solution to HIV acquisition or transmission, but also not providing a greater background on PrEP. We have now dropped the words, successful uptake, and highly effective solution. In addition, we provided some background context. The following amendment has been included in the background to address the concerns raised (page 4, lines 3-10).*

**In less than a decade, HIV pre-exposure prophylaxis (PrEP) has become a crucial component of global HIV prevention strategies, particularly for female sex workers (FSWs) and other populations at increased risk for HIV acquisition. In 2022, the relative risk of acquiring HIV was nine times higher for sex workers than in the wider adult (aged 15–49 years) population globally. Daily oral tenofovir and co-formulated emtricitabine-tenofovir disoproxil fumarate (FTC/TDF) have been shown to significantly reduce the incidence of HIV. Specifically, studies conducted among heterosexual men and women in Uganda and Kenya demonstrated a 67% reduction in HIV incidence with daily oral tenofovir, and a 75% reduction with co-formulated FTC/TDF**

2. Please describe why Mbale and Mbarara were selected for the research project within the introduction.

*Response: We thank the reviewer for this observation. We are sorry that in our submission we were unclear of why Mbale and Mbarara were selected. This sub-analysis was nested in a larger a quasi-experimental study that assessed the effect of peer education and text message reminders on the uptake of regular sexually transmitted infection (STI) and HIV testing among sex workers in Uganda. In the main study, Mbarara was the intervention site (peer education and short text mobile reminder messages), while Mbale was the control site. Prior to the quasi-experiment, FSWs in Mbarara and Mbale had low baseline syphilis and HIV testing intentions and practices between July and October 2018. We are unable to describe this in the background but rather in the methods section under study design and setting as we collected this data within a main study.*

3. Please describe the parameters used conducting the mapping exercise. What qualifies as a sex work hotspot?

*Response: We thank the reviewers for this observation. We apologize for not being clear. Otherwise, in our work a sex work hotspot meant locations frequented by sex workers or where sex work occurs. We have under the section on sampling and recruitment procedures described how the mapping was done, and what constituted a sex work hotspot. The text below has been added (page 6, lines 8-12);*

**A two-stage sampling design was used to recruit respondents. Prior to participant recruitment, a mapping exercise was conducted to gather insights into the dynamics of sex work in each city. In consultation with local health workers and barmaids, high volume sex work hotspots (locations frequented by sex workers or where sex work occurs) were enlisted**.

4. Please further describe the impact of this research within the discussion and conclusions.

*Response: We thank the reviewers for this recommendation. We are sorry our discussion was unable to be exhaustive. We have strengthened the discussion to include that the findings call for the need for awareness campaigns and implementation science studies. In particular the need to tailor the campaigns and studies to FSWs with low education levels.*

**Reviewer II**

**General comment**

This is an interesting and clearly written manuscript that describes PrEP use and associated factors among FSWs in two Ugandan cities

**Response:** *We thank the reviewers for this uplifting and encouraging comment.*

**Introduction:**

1. Avoid use of the word “solution”, which implies 100% effectiveness

*Response: We apologize the use of the term solution. We have now dropped it altogether from the current submission.*

2. Avoid use of the term “high-risk”, instead specify population at increased risk for HIV infection

*Response: We thank the reviewer for this observation and suggestion. We have adopted the suggestion of specifying the population or use the term population at increased risk for HIV infection throughout the manuscript.*

3. The authors should focus on FSWs, rather than high-risk population as a whole and then provide greater context regarding the role of FSWs in the HIV epidemic in SSA

*Response: We thank the reviewer for this guidance. We have now updated this section and provided the contribution for FSWs to HIV epidemic. The following text has been added (page 4, lines 13-18);*

**In 2022, an estimated 25% of new HIV infections in SSA were attributed to sex workers, their clients, and other populations at increased risk for HIV acquisition. Importantly, FSWs act as a bridge population - up to 15% of HIV infections in the general adult female population are attributable to sex work. In Uganda, FSWs exhibit a high HIV prevalence, ranging from 31% to 52%, and account for 7-11% of new HIV infections**.

4. Lines 7-8: This is misleading. A number of other populations at increased risk for HIV were included in the metanalysis.

*Response: We apologize for having cited this meta-analysis without a critical look. We have since dropped this reference, and focus on more specific references that focused on FSWs;*

**Faini D, Munseri P, Sandstrom E, Hanson C, Bakari M. Awareness, willingness and use of HIV pre-exposure prophylaxis among female sex workers living in Dar-es-Salaam, Tanzania. AIDS and Behavior. 2023;27(1):335-43.**

**Guure C, Afagbedzi S, Torpey K. Willingness to take and ever use of pre-exposure prophylaxis among female sex workers in Ghana. Medicine. 2022;101(5): e28798.**

**Witte SS, Filippone P, Ssewamala FM, Nabunya P, Bahar OS, Mayo-Wilson LJ, et al. PrEP acceptability and initiation among women engaged in sex work in Uganda: Implications for HIV prevention. EClinicalMedicine. 2022;44.**

**Nwagbo E, Ekwunife O, Mmeremikwu A, Ojide C. Awareness of and willingness to use pre-exposure prophylaxis to prevent HIV infection among female sex workers in Anambra State, south-eastern Nigeria. African Journal of Clinical.**

**Witte SS, Filippone P, Ssewamala FM, Nabunya P, Bahar OS, Mayo-Wilson LJ, et al. PrEP acceptability and initiation among women engaged in sex work in Uganda: Implications for HIV prevention. J EClinicalMedicine**

5. Lines 9-10: References 7 and 8 evidence PrEP acceptability rates of 91 and >50%. Not clear what data supports the 100% acceptability rates stated here

*Response: Thank you for this observation. We have now only focused on data that focused on FSW as per the references in 4 above.*

6. The authors should define PrEP persistence, and clarify the distinction made, if intentional between persistence and long-term use. They should also, clarify what is meant by diverse settings.

**Response: We apologize for the confusion caused. We have defined PrEP persistence to mean sustained use over time in this study. We also, agree with the reviewer long term use and diverse settings are confusing and have been deleted**.

7. The authors should clarify what is meant by everyday practice.

*Response: we apologize for not being clear. We had intended to mean PrEP adoption as a prevention intervention by FSWs. However, we have no deleted it altogether.*

Methods:

1. Line 15: The authors should clarify the language (s) in which the questionnaire was administered and proficiency of both parties in the language(s).

*Response: We appreciate the reviewers for this recommendation. The text below has been provided under data collection (page 6, lines 16-18);*

**Interviews were conducted by a trained team member fluent in the respondent’s preferred language (English, Luganda or Runyankore).**

**Results:**

1. Lines 15-17; Average number of clients per week may better be described as a behavioral factor than a demographic characteristic.

*Response: We thank the reviewer for this suggestion. We also, agree that is another context it could be presented as a behavioral factor. However, we believe for this current article (reality/phenomenon), presenting it under demographics describes the demand for their services*.

2. Line 21: Based on the data in Table 1, the percentage of FSWs with primary or secondary education in Mbale is 90% not 85%.

*Response: We apologize for the error. We had erroneously indicated 35 instead for 30 for secondary education, this has since been corrected in Table 1.*

3. Line 22: The authors should clarify that marital status was a significant difference between the two cities

*Response: We thank the reviewers for this observation and suggestion. We have now implemented the recommendation. The following text has been included (page 8, lines 3-5);*

**Marital status significantly differed between the cities, with most FSWs in Mbale identifying as single (61%) and most in Mbarara describing themselves as separated (54%).**

4. Line 4-6 (page7): The authors should include PEP awareness and usage. They should also, consider specifying syphilis testing and HIV rather than generalizing STI/HIV testing.

*Response: We are grateful to the reviewers for these suggestions. We have now implemented them. The text below has been included (page, 8 lines, 18-19 & 9-14);*

**Also, PEP awareness and use were lower in Mbale (awareness: 45%, use: 8%) compared to Mbarara (awareness: 66%, use: 15%)**

*Page 8, lines 9-14*

**Compared to Mbale, FSWs in Mbarara reported higher rates of recent syphilis and HIV testing. Lifetime syphilis testing was significantly higher in Mbarara (97%) than in Mbale (61%), with recent testing (within the past three months) also more common in Mbarara (87%) than in Mbale (26%). Although lifetime HIV testing rates were similar between the two sites (Mbarara: 97%, Mbale: 93%), recent HIV testing was more frequent in Mbarara (82%) than in Mbale (52%).**

**Discussion:**

1. Lines 17-18 (page 7): The authors should opt for more precise language than “generally low” to describe what are the principal findings of the study. Additionally, in the abstract, the authors describe PrEP use in this cohort to be “very low,” which is substantially different from how it is described here. The authors should be consistent with the language used to describe these findings.

*Response: we are sorry for the choice of language and inconsistence in reporting our findings. We have now revised both in the discussion and abstract to be more precise and consistent. In our interpretation both in the discussion and abstract we are only using low instead of very low. The text below has been included to replace general low (page 9, lines 5-6)*

**Less than half (42.3%) of the FSWs reported being aware of oral PrEP, with only 9.7% reporting current use.**

2. Lines 21-22 (page 7): The authors should specify the “services” to which they refer here.

*Response: We are sorry for being unclear. We have since corrected this to refer to PrEP services (page 9).*

4. Lines 22-4 (pp. 7-8): The authors should switch the order of these sentences (i.e., “Notably…” before “The differences in…”) to present the relevant findings prior to the speculative clause.

*Response: We thank the reviewers for this observation and suggestion. We have now implemented the recommendation (page 9, lines 11- 17)*

5. Lines 2-3 (page 8): The authors should provide a reference here for the PrEP demonstration project in Mbarara that is mentioned.

*Response: We are sorry for not providing this reference. We have now provided a reference for a PrEP project implemented at Mbarara regional referral hospital targeting women at increased risk of HIV infection*

**Matthews LT, Atukunda EC, Owembabazi M, Kalyebera KP, Psaros C, Chitneni P, et al. High PrEP uptake and objective longitudinal adherence among HIV-exposed women with personal or partner plans for pregnancy in rural Uganda: A cohort study. PLoS medicine. 2023;20(2):e1004088**.

6. Lines 4-5: The authors should refine this sentence (“Despite…”) to be more precise. This study assessed current usage of oral PrEP services among FSWs in two cities in Uganda. The findings, as written here, seem to refer to the current usage of oral PrEP services among the general population across all of Uganda.

*Response: We apologize for being unclear. We have revised the sentence and reference to FSWs (page 9, lines 19-20*).

7. Lines 5-6: This sentence needs to be reworked. The findings that current usage of oral PrEP services among FSWs was low do not directly “contrast” those of previous studies that evidence high willingness among FSWs to use oral PrEP; rather, they simply highlight that willingness to use (which is different from “intention,” and would make this change as well) and actual usage are two different things.

*Response: We are sorry for this confusion. We have now corrected the sentence to acknowledge the fact that willingness to use and actual use are not the same (page 10, lines 3-5)*

8. Lines 11-16: The authors should specify what is meant by “key populations” on lines 15-16 i.e., populations at increased risk for HIV? Additionally, the authors should consider including a reference or two that are specific to the population of interest (i.e., FSWs) if possible.

*Response: We thank the reviewers for this observation. We have now adopted the term “population at increased risk for HIV” throughout the manuscript. We have also, included references specific to FSWs*

**Faini D, Munseri P, Sandstrom E, Hanson C, Bakari M. Awareness, willingness and use of HIV pre-exposure prophylaxis among female sex workers living in Dar-es-Salaam, Tanzania. AIDS and Behavior. 2023;27(1):335-43.**

**Guure C, Afagbedzi S, Torpey K. Willingness to take and ever use of pre-exposure prophylaxis among female sex workers in Ghana. Medicine. 2022;101(5): e28798.**

**Witte SS, Filippone P, Ssewamala FM, Nabunya P, Bahar OS, Mayo-Wilson LJ, et al. PrEP acceptability and initiation among women engaged in sex work in Uganda: Implications for HIV prevention. EClinicalMedicine. 2022;44.**

**Nwagbo E, Ekwunife O, Mmeremikwu A, Ojide C. Awareness of and willingness to use pre-exposure prophylaxis to prevent HIV infection among female sex workers in Anambra State, south-eastern Nigeria. African Journal of Clinical.**

**Witte SS, Filippone P, Ssewamala FM, Nabunya P, Bahar OS, Mayo-Wilson LJ, et al. PrEP acceptability and initiation among women engaged in sex work in Uganda: Implications for HIV prevention. J EClinicalMedicine**

9. Lines 17-18: Again, the authors should consider specifying what is meant by “those who need it most” i.e., populations at increased risk for HIV.

*Response: We thank the reviewers for noticing this error on our part. We have now replaced this with FSWs (page 10 line 6 & 14)*

10. Lines 19-3 (pp. 8-9): The authors should include non-random sampling/ recruitment of survey respondents as a possible limitation to the study.

Response: We thank the reviewers for this suggestion. We have now implemented it (page 10 lines 20-21)

**Furthermore, peer referrals, a non-random sampling strategy was used to enrol study respondents.**

**Conclusions:**

1. The authors make sound conclusions and suggest practical next steps that follow from the data presented.

Response: We are grateful to the reviewers for this uplifting comment

**Table 2:**

2. The authors should consider unitalicizing the values in this table for uniformity with Tables 1 and 3. They should also consider adding n values for the individual variables listed

*Response: We are grateful to the reviewers for their suggestion. We have implemented the correction*

We thank the reviewers for their helpful comments which have improved the quality of our manuscript. We trust that the revised manuscript will be suitable for acceptance, but are happy to consider further revisions, if necessary.

Sincerely

Richard Muhindo, PhD, MPH, BSN

**Senior Lecturer**, Department of Nursing, College of Health Sciences

Makerere University

Corresponding author

Email: r.muhindo@yahoo.com
