# Supplementary material for: Associations with HIV preexposure prophylaxis use by cisgender female sex workers in two Ugandan cities
Source: PLoS One. 2025 Mar 20;20(3):e0320065. doi: 10.1371/journal.pone.0320065 (PMC11925456; doi:10.1371/journal.pone.0320065)
Supplement: Attachment — Submitted filename: Response_to_Reviewers_auresp_2.doc [file pone.0320065.s004.doc]

**Makerere	University**

P.O. Box 7072 Kampala, Uganda	**Tel:** 256 414 530404

**E-mail**: [nursing@chs.mak.ac.ug](mailto:nursing@chs.mak.ac.ug)

**SCHOOL OF HEALTH SCIENCES**

**Department of Nursing**

Dr. Hamufare D Mugauri

Academic Editor, Plos One

February 8^th^, 2025

Dear Dr. Hamufare,

Regarding PONE-D-24-24744

We are very grateful for the reviewer comments provided by the reviewers of our manuscript: “*Associations with HIV Preexposure Prophylaxis Use by Cisgender Female Sex Workers in Two Ugandan Cities ".* Reviewer comments are encouraging, and the reviewers appear to share our view that study findings are of public health importance. The suggestions offered by the reviewers have been greatly helpful. Please find below our point-by-point responses in italics, with new text in bold font:

**Reviewer Comments**

**Reviewer 3**

1. The authors present the results of an investigator-delivered survey administered to 236 FSW at risk for HIV in Mbale and Mbarara, Uganda in early 2020. The focus of the manuscript was use of oral TDF-based HIV PrEP. Overall knowledge of HIV PrEP and reported use (see below was low in a group of individuals with heighted risk of HIV acquisition. Data that sheds light into knowledge and use of PrEP in this group is worthy of attention. The differences by location and educational access in the adjusted models are interesting though not surprising.

*Response: We thank the reviewers for this encouraging overall comment regarding our work.*

2. The main issue with the manuscript is the lack of clarity around the primary outcome. It is not clear if this refers to current daily oral PrEP use, or ever use, or use in the prior 3 months. Please make it clear the time frame for PrEP use. In the abstract the authors describe current use.

*Response: We thank the reviewer for this observation. We are sorry for being unclear about the primary outcome. However, our primary outcome was current use/self-report of oral PrEP use at the time of data collection. Respondents were asked if they were currently taking daily pills to prevent HIV acquisitions. The text below has been added (page 6, line 17 & 20).*

**The primary outcome was current PrEP use, defined as self-reported use of daily oral pills to prevent HIV acquisition at the time of the interview.**

3. We are to assume that the regression is based on N=23. This means that the reference cell in the City variable in Table 3=0?

*Response: We thank the reviewers for this observation. True our regression is based current use, and therefore in a sample of 236, only 23 reported use at the time of data collection in Mbarara, we therefore thought our approach to model this data (treated as count data) using a modified Poisson regression, with the ability to account for any potential overdispersion or correlation in the data, providing more reliable inference was ok. However, we are open to suggestions to improve this.*

4. The other important issue is a more thorough explanation of differences by location in the discussion. This is important for readers unfamiliar with Uganda. Why might Mbarara have better awareness? Is it just that the demonstration project had increased awareness and use. What is the role of HIV prevalence, ongoing HIV-focused research that increase community-level awareness of HIV and HIV prevention.

*Response: We thank the reviewers for this recommendation. We have improved the explanation to capture the role of HIV prevalence, ongoing HIV focused research.* *The text below has been included (page 9, lines 9-17)*

**Our finding that FSWs in Mbarara were twice as likely to be aware of PrEP and report current use compared to those in Mbale highlights limited awareness and uptake in certain Ugandan urban locations. This disparity may be attributed to Mbarara's high adult HIV prevalence (14.4% vs 5.4% in Mbale), which has likely fueled ongoing HIV-focused research and increased community-level awareness of HIV prevention. Furthermore, the presence of a PrEP demonstration project targeting women at increased risk of HIV acquisition in Mbarara may also contribute to the observed difference**

5. Including the SAPPH-IRe trial would be useful

Response: We thank the reviewers for this recommendation. We have reviewed the trial found it very important as it evaluated a *dedicated programme/support for female sex workers in Zimbabwe accessing and adhering to antiretrovirals for treatment and prevention. From the article I reviewed, most of the FSWs in the trial were HIV positive, and with the intervention adherence and viral suppression was improved, I would have loved to include it but given that in this study, our focus was mainly to assess current PrEP use among HIV-naïve FSWs I wasn’t sure where to include it.*

6. Affiliations: What institution for the first author?

*Response: We apologize for not providing complete information on the institutional affiliations of the first author. This has been included. The following text has been included (title page)*

**^1^Department of Nursing Makerere University College of Health Sciences**

7. Abstract: Capital 'A' needed line 18

*Response: We thank the reviewers for noticing this error. We have since implemented the recommendation* on page 2, line 18

**Introduction**

1. Consider deleting 'in less than a decade' - PrEP FDA-approved in 2012. Or do you mean that the role of PrEP for FSW has only been understood for <10 years? The SAPPH-IRe trial was 2014-16.

*Response: We thank the reviewer for this recommendation. We have since implemented the recommendation on page 4, line 2*

2. Page 4. Lines 13-15. Are there specific data on role of FSW? The specific data are 'hidden' in the total of the KP numbers. It would be much better here to cite the original ref (Korenromp et al 2024) and give the data on partners of SW - table 2, rather than a composite).

*Response: We thank the reviewers for this suggestion. We have since implemented it in the introduction (page 4, line 13). The text below referenced to Korenromp et al 2024 has been included*.

**In 2022, sex workers accounted for an estimated 5.2-15% of new HIV infections in Sub-Saharan Africa**

3. Page 4. Lines 16-18 provides better information than lines 13-15. Suggest sing this instead

*Response; We thank the reviewer for this encouraging comment. We have revised lines 13-15 to provide information that focuses on sex workers on page 4, line 13-17*

4. Page 5. Lines 4-11. This is interesting but does not really relate to the described outcomes which describe knowledge and use. Not persistence. I would suggest shortening this. It gives the impression that this is the focus of your analysis.

*Response: We thank the reviewer for this observation. We have implemented the suggestion by deleting persistence and focus on use (page 5, lines 4-9)*

Methods:

1. Page 6. Lines 1-2. What does that mean? What was the baseline HIV testing uptake during that time period compared with 2020? Important context. The 2019 AIDS Res Ther showed that 67.4% of FSW took at least 2 HIV tests in the prior 12 months. Which is essentially the same as you report here.

*Response: We sorry if our statement regarding the rationale to select Mbarara and Mbale was unclear. While our BMC Health Serv Res 21, 436 (2021).* [*https://doi.org/10.1186/s12913-021-06461-w*](https://doi.org/10.1186/s12913-021-06461-w) *article gives a better picture of the changes between 2018 and 2020, we have removed the statement and indicated that Mbale and Mbarara served as control and intervention sites for our quasi-experiment, and as such were purposely selected for the PrEP use study (page 5, lines 17-19). The text below has been included*

**Mbarara and Mbale served as the intervention and control sites, respectively, for our quasi-experimental study, and were purposively selected for the PrEP use survey**.

2. Sex work hotspots - page 6. Line 6. Move up the explanation from lines

10-11.

*Response: we thank the reviewers for this recommendation. We have implemented as suggested* (pages 5, line 22, and page 6, lines 1-10)

3. Can you explain what expertise barmaids have? This will not be clear to some readers in places where barmaid work is not related to sex work knowledge.

*Response: We thank the reviewers for this observation. We have included the text below to implement this recommendation (page 6, line 4)*

**In Uganda, barmaids may engage in sex work or facilitate connections between sex workers and clients**

4. Page 6. Lines 21-22. Page 7. Lines 2-3. Per the comment above is this current or ever use? What is the timeframe? I assume from the abstract that it is current use, it would be good to make this very clear.

*Response: We apologize for being unclear. We have since consistently referred to current use, defined as self-reported use of daily oral pre-exposure prophylaxis (PrEP) pills to prevent HIV acquisition at the time of the interview throughout the article* (page 6, lines 17 & 20)

5. Page 7. Lines 11-15. Were the participants compensated?

*Response: We are sorry for not stating this. However, all participants were compensated for their time*. The text below has been included (page 7, lines 11-12)

**All respondents provided written informed consent in English or their local language, and were compensated for their time**.

Discussion:

1. Page 9. Line 7. is it correct to say 'three times' Range was 2.5-3.1? Maybe 2.5-3x.

*Response: We thank the reviewers for this suggestion. We have since implemented the recommendation (page 9, line 5)*

2. Page 9. Lines 16-17. This seems somewhat tangential as is HIV-focused. This needs some more explanation if it is to stay in. Do the authors mean that physical location is the determining factor or transportation, educational activities, presence of NGOs etc wrt to HIV services?

Response: We apologize for this error on our part. We have since revised to highlight the role of ongoing research and programs. *The text below has been included (page 9, lines 10-18)*

**Our finding that FSWs in Mbarara were twice as likely to be aware of PrEP and report current use compared to those in Mbale highlights limited awareness and uptake in certain Ugandan urban locations. This disparity may be attributed to Mbarara's high adult HIV prevalence (14.4% vs 5.4% in Mbale), which has likely fueled ongoing HIV-focused research and increased community-level awareness of HIV prevention. Furthermore, the presence of a PrEP demonstration project targeting women at increased risk of HIV acquisition in Mbarara may also contribute to the observed difference**

3. Page 9. Line 19. Again, clarify the 'when' of PrEP use.

*Response: We sorry, and thank the reviewers for noticing this. We have since consistently used current use in the manuscript.*

4. Some of the discussion is repetitive. For example, the authors could make more succinct by combining. Page 9. Lines 20-21 with Lines 9-10. Page 10. Lines 5-6. Similar to lines 9-10, 20-21. Could combine all of these. Page 10. Lines 7-9 are very similar to Page 9. Lines 7-9. Could combine.

*Response; We are sorry for this error. We have implemented the recommended and now the discussion goes as below (page 9, lines 1-23*

**This cross-sectional study investigated current oral PrEP use among FSWs in two Ugandan cities. Less than half (42.3%) of the FSWs reported being aware of oral PrEP, with only 9.7% reporting current use. However, awareness and use significantly varied by location. Notably, FSWs who had attained secondary or tertiary education were 2.5-3 times more likely to report current use, consistent with previous studies conducted in SSA (29-31). These studies have consistently shown a positive correlation between education level and PrEP usage, highlighting the need for implementation research on strategies to promote PrEP use among FSWs with lower education levels.**

**Our finding that FSWs in Mbarara were twice as likely to be aware of PrEP and report current use compared to those in Mbale highlights limited awareness and uptake in certain Ugandan urban locations. This disparity may be attributed to Mbarara's high adult HIV prevalence (14.4% vs 5.4% in Mbale) (10), which has likely fueled ongoing HIV-focused research and increased community-level awareness of HIV prevention (33, 34). Furthermore, the presence of a PrEP demonstration project targeting women at increased risk of HIV acquisition in Mbarara may also contribute to the observed difference (34). Targeted educational campaigns to FSWs regarding the benefit and availability of oral PrEP services are still needed.**

**Regional variations in PrEP usage have been observed among FSWs in East Africa. While our finding of low current PrEP use (9.7%) is comparable to a Kenyan study (24%) (29), a recent Tanzanian study reported notably higher use (97%) (30). These disparities likely stem from contextual differences in service organization, provider characteristics, and recipient factors. Existing literature cites various barriers to non-oral PrEP use among FSWs, including limited access, lack of awareness, negative perceptions, stigma, and pill burden (29, 38, 39). Despite these insights, targeted interventions are still necessary to improve PrEP uptake and usage.**

**This cross-sectional study, conducted prior to the first COVID-19 lockdown in Uganda, provides a snapshot of PrEP use among FSWs in Mbale and Mbarara. However, the findings may not capture trends in PrEP use over time. Additionally, self-reported PrEP use may be subject to social desirability bias. Moreover, the study's sample was limited to two cities and may not be generalizable to all FSW communities in Uganda. Furthermore, peer referrals, a non-random sampling strategy was used to enroll study respondents. Despite these limitations, the results highlight the need for targeted educational campaigns and implementation studies to increase PrEP uptake among FSWs, particularly in areas with low awareness and usage beyond demonstration projects.**

**Conclusions**

**The low PrEP usage in this FSW cohort underscores the need for implementation studies that evaluate strategies to improve uptake, particularly among those with lower levels of education.**

5. The authors mention 'willingness' on page 10, lines 3-4. You did not measure willingness in your population, correct? I would make clear that you are assuming willingness in other studies equates to willingness in FSW in Mbale and Mbarara.

*Response: We are sorry for this error. We have now referenced to only use, page 9, lines 19-23, page10, lines 1-3).*

Conclusions:

1. Page 11. Lines 5-8. This is essentially repetition of lines 21-22 on page 10.

*References: we are sorry. We have amended as follows (page 10, line 15)*

**The low PrEP usage in this FSW cohort underscores the need for implementation studies that evaluate strategies to improve uptake, particularly among those with lower levels of education**

#9. I cannot find the data referenced in the text when I click on this link. Why are the authors using 2021 Ugandan fact sheet? Are there more recent data available?

Response: *We are sorry that the provided link is unable to open. We have provided a better link; Uganda AIDS Commission. HIV Factsheet 2023. FACTS ON HIV AND AIDS IN UGANDA 2023 (Based on Data Ending 31st December 2022) [accessed 2025 February] Available from;* [*https://uac.go.ug/index.php 2023*](https://uac.go.ug/index.php 2023)*. The most recent data is 2024 but doesn’t provide data on FSWs. We have however, used the data of 2022 instead of 2021.*

#10. Are there no more recent data than 2009?

*Response: Sorry, we used this study, because the 2009 modes of transmission study estimated the contribution of sex work to new HIV infections. While some articles (Witte, Susan S., et al. "PrEP acceptability and initiation among women engaged in sex work in Uganda: Implications for HIV prevention." EClinicalMedicine 44 (2022)), have quoted Uganda AIDS commission fact sheets 2020, review of this fact sheet doesn’t show any information related to the contribution of sex work to new HIV infections.*

Tables:

All need a key with abbreviations spelled out.

*Response: we thank the reviewers for this suggestion. We have implemented the recommendation (pages 19, line2; page 20, line 2; page 21, line 2)*

Table 1. Typology is ambiguous here. Suggest 'sex work venue' or similar.

*Response: We thank the reviewer for this suggestion.*

Escort is a type of SW not a venue.

*Response: We thank the reviewers for noticing this error. We have revised.*

Table 2.

Ever hear - add 'of PEP'.

*Response: Thank you for noticing this error (page 20, line 2)*

0.90 - text says 97.

0.81 - text says 87.

*Response: We thank the reviewer for noticing this error where we mixed syphilis with HIV (page 8, line 7)*

**Reviewer 4**

1. The manuscript highlights a critical and timely issue, as female sex workers (FSWs) remain a population of focus for HIV prevention efforts across sub-Saharan Africa (SSA). The gaps in accessing effective prevention services within this population underscore the significance of the study's findings and the contribution it could make to the field.

*Response: We thank the reviewer for this uplifting comment*

2. The authors have commendably addressed most of the concerns raised by the previous reviewers, demonstrating their commitment to improving the manuscript. However, several issues still require attention to enhance the clarity, rigor, and overall impact of the paper.

*Response: We thank the reviewer for highlighting more areas that could improve our manuscript*

3. The aim and study design:There are inconsistencies in the study aim, design, and outcome as presented across different sections of the manuscript. Abstract: In a quasi-experimental study to assess the effect of peer education and text message reminders on the uptake of regular sexually transmitted infection (STI) and HIV testing, we administered a survey questionnaire to 236 HIV-negative FSW in the cities of Mbale and Mbarara from January to March 2020Introduction: Page 4, lines 19-21 This study aimed to assess PrEP use and associated factors among FSWs in two urban settings in Uganda, providing valuable insights into PrEP program implementation in everyday practice

*Response: We apologize for this inconsistency. We have throughout the manuscript we consistently revised the outcome to refer to self-report use of oral PrEP at the time of data collection. We have also, revised the study aim in relation to the aim i.e. current oral PrEP use and associated factors. Additionally, our study design both in the abstract and methods now shows that this was a cross-sectional survey nested in a quasi-experimental study. Text below has been included in the methods (page 6, lines 17 & 20)*

**The primary outcome was current PrEP use, defined as self-reported use of daily oral pills to prevent HIV acquisition at the time of the interview**

*Also, study aim (page 5, line 8)*

**This study aimed to assess current oral PrEP use and associated factors among FSWs in two Ugandan cities**.

*Additionally, study design (page 2, line 6)*

**We administered a survey questionnaire to 236 HIV-negative FSW in the cities of Mbale and Mbarara from January to March 2020. The survey was nested in a quasi-experimental study to assess the effect of peer education and text message reminders on the uptake of regular sexually transmitted infection (STI) and HIV testing**

4. The authors have indicated that the primary outcome is PrEP use, defined as self-reported use of daily oral pills. However, it is unclear whether they meant use at the time of the interview, or for a particular period. In the discussion, the phrase ‘current use’ is used repeatedly. Providing a clearer definition will ensure consistent understanding among diverse readers

*Response: We thank the reviewer for this observation. We are sorry for being unclear about the primary outcome. However, our primary outcome was current use/self-report of oral PrEP use at the time of data collection. Respondents were asked if they were currently taking daily pills to prevent HIV acquisitions. The text below has been added (page 6, line 17 & 20).*

**The primary outcome was current PrEP use, defined as self-reported use of daily oral pills to prevent HIV acquisition at the time of the interview.**

5. Study setting:While the authors have addressed the previous reviewer’s question regarding the selection of Mbale and Mbarara for this study, the explanation provided in the methods section remains unclear. Please review this section to ensure the rationale is clearly articulated

*Response: we are sorry for this error on our part. We have revised to state that Mbarara and Mbale served as intervention and control sites, respectively for our quasi-experimental study, and were and were purposively selected for this survey. We have also, cited this study (page 5, line 17-19)*

*Muhindo R, Mujugira A, Castelnuovo B, Sewankambo NK, Parkes-Ratanshi R, Kiguli J, et al. Text message reminders and peer education increase HIV and Syphilis testing among female sex workers: a pilot quasi-experimental study in Uganda. BMC health services research. 2021;21(1):436.*

6. Discussion:
   While the authors discuss the main findings, the writing needs to be tightened for more coherence and clarity including appropriate citations. e.g the discussion of the correlation between education level and PrEP uptake. In addition, the authors have made a comparison between their findings and those of studies evaluating willingness to use PrEP, while there are a lot of publications on PrEP uptake among FSW in similar settings.

*Response: We apologize for this we have revised the discussion, and referenced only citations on PrEP use (pages 9 & 10)*

**This cross-sectional study investigated current oral PrEP use among FSWs in two Ugandan cities. Less than half (42.3%) of the FSWs reported being aware of oral PrEP, with only 9.7% reporting current use. However, awareness and use significantly varied by location. Notably, FSWs who had attained secondary or tertiary education were 2.5-3 times more likely to report current use, consistent with previous studies conducted in SSA (29-31) . These studies have consistently shown a positive correlation between education level and PrEP usage, highlighting the need for implementation research on strategies to promote PrEP use among FSWs with lower education levels.**

**Our finding that FSWs in Mbarara were twice as likely to be aware of PrEP and report current use compared to those in Mbale highlights limited awareness and uptake in certain Ugandan urban locations. This disparity may be attributed to Mbarara's high adult HIV prevalence (14.4% vs 5.4% in Mbale) (10), which has likely fueled ongoing HIV-focused research and increased community-level awareness of HIV prevention (33, 34). Furthermore, the presence of a PrEP demonstration project targeting women at increased risk of HIV acquisition in Mbarara may also contribute to the observed difference (34). Targeted educational campaigns to FSWs regarding the benefit and availability of oral PrEP services are still needed.**

**Regional variations in PrEP usage have been observed among FSWs in East Africa. While our finding of low current PrEP use (9.7%) is comparable to a Kenyan study (24%) (29), a recent Tanzanian study reported notably higher use (97%) (30). These disparities likely stem from contextual differences in service organization, provider characteristics, and recipient factors. Existing literature cites various barriers to non-oral PrEP use among FSWs, including limited access, lack of awareness, negative perceptions, stigma, and pill burden (29, 38, 39). Despite these insights, targeted interventions are still necessary to improve PrEP uptake and usage.**

**This cross-sectional study, conducted prior to the first COVID-19 lockdown in Uganda, provides a snapshot of PrEP use among FSWs in Mbale and Mbarara. However, the findings may not capture trends in PrEP use over time. Additionally, self-reported PrEP use may be subject to social desirability bias. Moreover, the study's sample was limited to two cities and may not be generalizable to all FSW communities in Uganda.** **Furthermore, peer referrals, a non-random sampling strategy was used to enroll study respondents. Despite these limitations, the results highlight the need for targeted educational campaigns and implementation studies to increase PrEP uptake among FSWs, particularly in areas with low awareness and usage beyond demonstration projects.**

***Conclusions***

**The low PrEP usage in this FSW cohort underscores the need for implementation studies that evaluate strategies to improve uptake, particularly among those with lower levels of education.**

We thank the reviewers for their helpful comments which have improved the quality of our manuscript. We trust that the revised manuscript will be suitable for acceptance, but are happy to consider further revisions, if necessary.

Sincerely

Richard Muhindo, PhD, MPH, BSN

**Senior Lecturer**, Department of Nursing, College of Health Sciences

Makerere University

Corresponding author

Email: r.muhindo@yahoo.com
